# Clinical and cognitive factors associated to insight in first psychotic episodes

**DOI:** 10.1192/j.eurpsy.2024.1551

**Published:** 2024-08-27

**Authors:** T. Gonzalez Salvador, I. Cabello Rojano, S. Boi, R. Gutierrez Labrador, P. R. Capilla

**Affiliations:** ^1^Psiquiatría, Hospital Universitario Puerta de Hierro; ^2^Medicina Interna, Hospital Infanta Sofía; ^3^Psiquiatría, Hospital Universitario Infanta Sofía; ^4^Psiquiatría, Hospital La Paz, Madrid, Spain

## Abstract

**Introduction:**

Insight is a field of interest in psychosis, due to its influence on the course and prognosis of the desease and as well as adherence to treatment.

**Objectives:**

The present work aims to evaluate the influence of cognitive and psychopathological variables on awareness of illness in first psychotic episodes.

**Methods:**

It is a cross-sectional study of a sample of 26 patients with diagnosis of a first psychotic episode admitted in a Brief Hospitalization Unit, who have been evaluated using the Positive and Negative Symptom Scale (PANSS), the Screening for Cognitive Impairment (SCIP) and the Scale of Non-awareness of Mental Disorder (SUMD).

**Results:**

A positive correlation was found between SUMD and negative PANSS (the worse insight, the greater negative psychopathology) and between the level of cognitive performance and the awareness of having negative symptoms (affective blunting, anhedonia and associability) and their attribution to the desease.

**Conclusions:**

This findings suggest the importance of addressing awareness of negative symptoms from the first episodes in psychoeducational family therapy and rehabilitation programs, taking into account that this process is hindered by the cognitive dysfunctions.

**Disclosure of Interest:**

None Declared

